# EEG-Based Emotion Recognition with Consideration of Individual Difference

**DOI:** 10.3390/s23187749

**Published:** 2023-09-08

**Authors:** Yuxiao Xia, Yinhua Liu

**Affiliations:** 1 College of Automation, Qingdao University, Qingdao 266071, China; xiayuxiao_55@163.com; 2 Insititute for Future, Qingdao University, Qingdao 266071, China

**Keywords:** EEG, emotion recognition, individual differences

## Abstract

Electroencephalograms (EEGs) are often used for emotion recognition through a trained EEG-to-emotion models. The training samples are EEG signals recorded while participants receive external induction labeled as various emotions. Individual differences such as emotion degree and time response exist under the same external emotional inductions. These differences can lead to a decrease in the accuracy of emotion classification models in practical applications. The brain-based emotion recognition model proposed in this paper is able to sufficiently consider these individual differences. The proposed model comprises an emotion classification module and an individual difference module (IDM). The emotion classification module captures the spatial and temporal features of the EEG data, while the IDM introduces personalized adjustments to specific emotional features by accounting for participant-specific variations as a form of interference. This approach aims to enhance the classification performance of EEG-based emotion recognition for diverse participants. The results of our comparative experiments indicate that the proposed method obtains a maximum accuracy of 96.43% for binary classification on DEAP data. Furthermore, it performs better in scenarios with significant individual differences, where it reaches a maximum accuracy of 98.92%.

## 1. Introduction

Emotion encompasses a variety of subjective cognitive experiences, playing a crucial role in human emotional experiences, and is essential for expression and feedback [1]. Emotions have significant implications in decision-making, and individuals experiencing prolonged negative emotions may undergo various negative physiological and psychological effects, such as immune system suppression, physical fatigue, sleep disorders, anxiety, and social isolation [2,3].

Emotion recognition involves estimating human emotions [4]. Emotions are typically expressed through subjective descriptions, physiological responses, and behavioral manifestations [5]. Among these, physiological signals are considered to provide more accurate representations of an individual’s emotions compared to facial expressions, vocalizations, and other modalities, as they are less influenced by subjective factors [6]. EEG signals are obtained by placing electrodes on the scalp to record brain electrical activity. An increasing number of researchers have begun to utilise EEG signals for classifying and recognising emotions.

Currently, EEG-based emotion recognition models can be broadly classified into two types: discrete models and dimensional models [7]. Discrete models classify emotions into several distinct categories, such as “happy”, “sad”, “angry”, etc. While these models are relatively simple and easy to implement, they fail to capture the complexity and diversity of emotions. Dimensional models view emotions as continuous dimensions, with each dimension representing a specific aspect of emotions. Russell’s bipolar dimensional model, which includes valence and arousal dimensions, is widely used in emotion recognition tasks [8]. Although these models are relatively complex, they can enhance the accuracy and precision of emotion recognition. In this study, we adopt a dimensional model. In recent years, deep learning techniques have been widely applied in EEG-based emotion recognition. Deep learning models can effectively learn and extract spatial and temporal features [9] from EEG signals, thereby improving the accuracy of emotion classification. Early studies primarily employed traditional shallow neural networks, decision trees, support vector machines (SVM), and other methods [10]. While these methods can achieve a certain degree of emotion classification, they are limited by their feature extraction and model design, leaving room for improvement. Qazi et al. [11] proposed a lightweight pyramidal one-dimensional convolutional neural network (LP-1D-CNN) model which automatically extracts and selects features for automatic emotion recognition using EEG signals. They analyzed the time–frequency domain features of raw EEG signals and explored inter-channel correlation information. Chen et al. [12] proposed a deep convolutional neural network (CNN) emotion recognition model based on the time–frequency dimension of input signals and used the combination of multiple features to improve recognition accuracy, demonstrating higher recognition accuracy and stability compared to traditional classifiers. Zhang et al. [13] proposed a spatio-temporal recursive neural network (STRNN) for emotion recognition, and their results showed that STRNN outperformed SVM. Alhagry et al. [14] proposed a network for emotion recognition from raw EEG signals, utilizing a long short term-memory recursive neural network (LSTM-RNN) to learn features from EEG signals and perform classification. Wei et al. [15] proposed an attention-based convolutional recurrent neural network (ACRNN) to extract discriminative features and obtain more information.

Dai et al. [16] proposed an HS-CNN model with a hybrid convolutional scale for EEG motor image classification; this hybrid-scale CNN architecture was able to achieve high classification accuracy. Li et al. [17] introduced a multidimensional approach based on the continuous wavelet transform and the Clough–Tocher interpolation algorithm for processing motor intention electroencephalography (MI-EEG) signals combined with a multilevel and multiscale feature fusion convolutional neural network (MLMSFFCNN) for recognition. Zhu et al. [18] proposed an emotion recognition method considering multi-band EEG data inputs based on a dynamic Simplified Graph Convolution (SGC) network and a channel-style recalibration module. Zhang et al. [19] proposed the idea of assigning channel weight ratios to the channels that are more strongly correlated with emotion. By using strong emotion correlation channels to assign large weights, their method was able to achieve recognition rates of 90.05% and 90.84%, respectively, in terms of potency and arousal.

Extensive research has focused on establishing emotion recognition models with high accuracy. However, these models rely on a large amount of EEG data from individual participants, and are only effective for the specific participants. Chen et al. [20] proposed the individual personal z-score (PZ) feature handling method to reduce the impact of individual differences and improve the accuracy of their emotion recognition model. Li et al. [21] applied experiment-level batch normalization (BN) to feature extraction for candidate sets and used normalized features for cross-individual emotion recognition. Zhang et al. [22] constructed user-dependent models using a small amount of training EEG data and proposed an individual similarity-guided transfer learning approach for EEG-based emotion recognition. Based on the framework of transfer learning, this method trained an emotion recognition model specific to the user and achieved a 70% accuracy rate. Koelstra et al. [23] applied transfer learning using two publicly available databases with different experimental paradigms, with the results demonstrating the challenges involved in transfer learning across different participants.

In summary, individual differences pose a significant challenge in the field of emotion recognition. Under the same external emotional stimuli, there exist substantial individual differences in the intensity of emotions and response time which can negatively impact the accuracy of emotion recognition. Therefore, it is crucial to reduce the influence of individual differences and establish an emotion recognition model that performs well across different participants. The contributions of this work can be summarized as follows:

1. The main contribution of this study is the development of an individual emotion recognition model (IERM) designed to handle cross-participant emotion classification tasks. The proposed model incorporates attention mechanisms after each convolutional layer in the CNN module, enhancing inter-channel relationships in EEG signals and leading to improved emotion recognition accuracy and reliability. The IERM is trained on well-established datasets, enabling the establishment of a generalized emotion recognition model that performs effectively across diverse participants.
2. To address the impact of individual differences on emotion recognition, an innovative IDM is integrated into the generalized emotion recognition model. The IDM optimizes classification results to better accommodate individual emotional responses during cross-participant emotion classification tasks, effectively reducing the influence of individual differences and enhancing the model’s ability to handle variations in emotional response among different individuals.

The rest of this paper is organized as follows: Section 2 presents the IERM proposed in this study; Section 3 describes the specific implementation of the model; Section 4 introduces the data preprocessing and experimental process of the IERM structure, including a performance evaluation of the algorithm; and Section 5 provides a summary of the proposed model.

## 2. Methods

**Figure 1:**
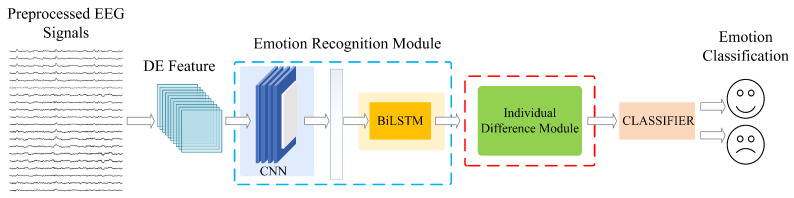
The proposed IERM framework.

The IERM proposed in this paper aims to mitigate the impact of individual differences. It comprises two components, namely, an emotion classification module and an IDM. The emotion classification module consists of a spatial domain module and temporal domain module. The spatial domain module incorporates manually extracted time–frequency domain differential entropy (DE) features and uses CNN-extracted spatial features as emotional features [24]; additionally, a channel attention mechanism layer is introduced into the hierarchical structure of the CNN. The temporal domain module utilizes bidirectional long short-term memory (BiLSTM) to extract temporal features, and employs a fully connected layer at the final layer of the model to classify the output of the BiLSTM layer. By considering both the temporal and spatial domains of EEG signals, this general emotion classification module can learn universal patterns of emotion classification from a large amount of data, effectively integrating the temporal and spatial characteristics of EEG signals for accurate emotion classification. The construction of the proposed model is shown in Figure 1.

The IDM aims to optimize emotion classification performance across subjects. Due to inter-individual differences, using a generic emotion classification module alone cannot accurately classify emotions for each individual. Therefore, we introduce the IDM on top of the emotion classification module. The IDM fine-tunes the emotion classification module and adjusts the specific emotional features for each subject individually, further improving the emotion classification performance.

### 2.1. Spatial Features

EEG feature extraction aims to discover the underlying discriminative features for each emotion and utilizes these features in the process of emotion classification and recognition. First, to extract the dynamic temporal information of EEG comprehensively and increase the amount of training data, the original EEG information, denoted as *S*, is divided into long non-overlapping segments. Each segment is assigned the label of the original trial, i.e., $S=\left\{X_{1},X_{2},\cdots ,X_{T}\right\}\in R^{\left(M\times N\right)}$, where *M* and *N* represent the number of electrodes and the number of sampling points, respectively. Previous studies [25,26] have shown four bands to be suitable for emotion classification based on EEG: θ (4–7 Hz), α (8–13 Hz), β (14–30 Hz), and γ (31–50 Hz). Therefore, the DE features of these four frequency bands were extracted for each segment. The formula is provided below.

(1) $$\begin{array}{cc} DE & =\int_{a}^{b}p\left(x\right)\log\left[p\left(x\right)\right]dx\\ \; & =\frac{1}{2}\log\left(2\pi e\delta^{2}\right)\int_{-\infty }^{+\infty }\frac{1}{\sqrt{2\pi \delta^{2}}}e^{\frac{p-\mu }{2\delta^{2}}}\log\left(\frac{1}{\sqrt{2\pi \delta^{2}}}e^{\frac{p-\mu }{2\delta^{2}}}\right)dp \end{array}$$

The probability density function of the continuous information is denoted as p(x), and follows a Gaussian distribution $N(\mu ,\delta^{2})$, while [a,b] represents the range of values for the information. For a specific length of EEG segment, its differential entropy is equal to the logarithm of the energy spectrum in a specific frequency band.

**Figure 2:**
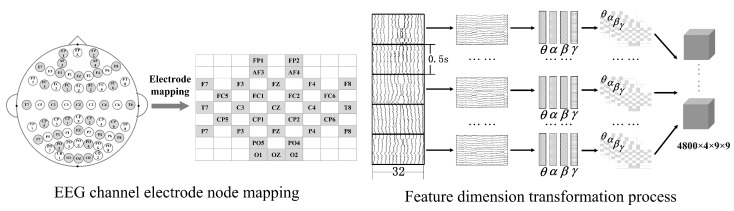
EEG feature dimension transformation.

Using the international 10–20 system, the DEAP data acquisition system comprises a total of 32 channels. Figure 2 illustrates the electrode mapping and feature dimension transformation processes within the EEG channels. To preserve the spatial structural information of the EEG electrode positions, a mapping process was employed based on the spatial coordinates of the EEG electrodes and their relative positions, resulting in a two-dimensional grid with dimensions of 9×9, which effectively translates the intricate three-dimensional electrode relationships into space. The DE features for each frequency band were extracted individually and assembled into a two-dimensional feature map according to the mapping principles outlined in Figure 2. These features map were further integrated by stacking those from four frequency bands to construct a three-dimensional feature map of size 4×9×9. The number of videos watched by each subject was 40, the duration of each video was 60 s, and each video was divided into several time windows according to the rule that every 0.5 s the EEG signals were equally divided into 4800 (40×60×0.5) segments. Three-dimensional features were then computed for each segment, resulting in a four-dimensional feature space with dimensions of 4800×4×9×9.

**Figure 3:**
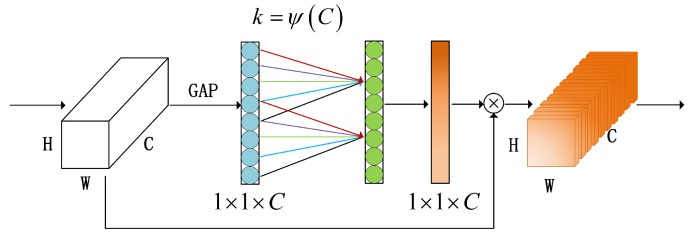
Structure of the ECA.

The structural diagram of the ECA mechanism is shown in Figure 3. For detailed mathematical explanations, please refer to reference [27]. This structure can mainly be divided into three aspects:

1. A squeeze operation is performed on the feature maps to achieve global context information fusion [28]. This step involves global average pooling (GAP) to transform the feature maps from size (N,C,H,W) to size (N,C,1,1), where *C* represents the number of input channels and *N*, *H*, and *W* respectively represent the number, height, and width of the convolution block.
2. The adaptive convolution kernel size is computed using the equation $k=\psi \left(C\right)=\left|\frac{\log_{2}C+b}{\gamma }\right|$, where b=1 and γ=2. One-dimensional convolution is then applied to calculate the channel weights and the sigmoid activation function is used to map the weights between 0 and 1.
3. The reshaped weight values are multiplied element-wise with the original feature maps (this step utilizes Python’s broadcasting mechanism [29]) to obtain feature maps with different weights.

**Figure 4:**
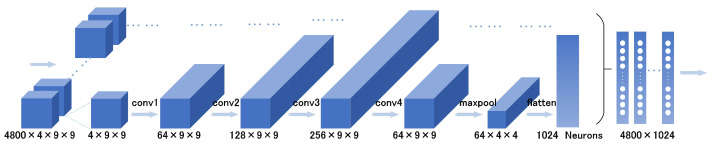
The CNN network architecture.

By incorporating the ECA mechanism layer after each convolutional layer in the CNN module, the representation capability of crucial channels is strengthened, enabling the network to emphasize key channels during emotion classification. Additionally, the ECA mechanism enhances interaction and information transfer among diverse channels in the convolutional layers, facilitating better capture of subtle features and patterns in the input data. By reducing attention on noise and redundant information, it enhances the network’s focus on effective features, thereby reducing interference and improving the accuracy and robustness of emotion classification. The ECA mechanism enhances the generalization ability of model generalization ability by adaptively learning channel weights, making it suitable for emotion classification tasks involving different individuals and real-world environments. This improved capability can contribute to the advancement of EEG emotion classification performance. The architecture of the CNN network is depicted in Figure 4. For a detailed explanation, please refer to Section 3.

### 2.2. Temporal Features

EEG signals are multi-dimensional time series data that carry spatial information and rich temporal dynamics. To better exploit this temporal information, the proposed model incorporates BiLSTM [30], a bidirectional recurrent neural network that extends the traditional LSTM structure by adding backward sequence processing. BiLSTM can simultaneously consider past and future contextual information, capturing long-term dependencies in sequences through both forward and backward propagation. This bidirectional processing enables BiLSTM to better understand the overall sequence context and make more accurate predictions. BiLSTM is robust against noise, missing data, and input variation, and adapts well to different input conditions. In addition, BiLSTM effectively addresses the gradient vanishing and exploding problems commonly encountered in traditional recurrent neural networks (RNNs), enabling it to capture long-term dependencies while improving the performance and stability of the model. The combination of the CNN network and BiLSTM layer allows the model to exploit both spatial and temporal information for comprehensive exploration of emotion-related features in EEG signals. The overall structural diagram is illustrated in Figure 4. The features extracted by the CNN are denoted as $H_{n}=\left(h_{1},h_{2},h_{3}\cdots ,h_{T}\right)$, where $h_{i}$ represents the features of the *i*th segment, while the output of the LSTM is denoted as $y_{n}$. The computation formulas for LSTM units are as follows.

(2) $$\begin{array}{c} i_{t}=\sigma \left(W_{i}\left[h_{t-1},x_{t}\right]+b_{i}\right)\\ f_{t}=\sigma \left(W_{f}\left[h_{t-1},x_{t}\right]+b_{f}\right)\\ {\tilde{C}}_{t}=\tanh\left(W_{C}\left[h_{t-1},x_{t}\right]+b_{C}\right)\\ C_{t}=f_{t}⨀c_{t-1}+i_{t}⨀{\tilde{C}}_{t}\\ o_{t}=\sigma \left(W_{o}\left[h_{t-1},x_{t}\right]+b_{o}\right)\\ h_{t}=o_{t}\tanh\left(c_{t}\right) \end{array}$$

In the above formulas, $f_{t}$ is the forgetting gate that filters out less important information, $i_{t}$ is the input gate that inputs effective information, $o_{t}$ is an output gate that outputs information, σ represents the sigmoid activation function, *W* represents the weight matrix corresponding to each gate, *b* represents the corresponding bias coefficient, $h_{t}$ and $h_{t-1}$ represent the respective outputs of the previous and current time steps, $x_{t}$ represents the input of the current time step, ${\tilde{C}}_{t}$ represents the candidate memory state, and $C_{t}$ represents the memory state of the cells of the previous and current time steps.

**Figure 5:**
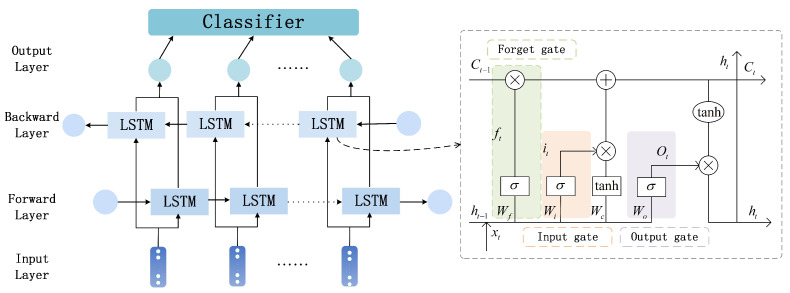
Temporal feature learning.

The linear layer serves as a classifier, mapping the output of BiLSTM to the prediction results of a binary classification problem. The model employs a threshold classifier (sigmoid function) to convert probabilities into the final binary labels. When treating the output as probability estimates, the mean squared error (MSE) is chosen as the loss function. By minimizing the MSE loss function, the model aims to reduce the difference between predicted values and actual values as much as possible [31]. The diagram of the overall architecture is illustrated in Figure 5. The calculation formula for each class is shown below.

(3) $$\begin{array}{c} P\left[c|g\left(x_{n},c\right)\right]=F_{\mathrm{sigmoid}}\left[g_{i}\left(x_{n},c\right)\right]=\frac{1}{1+e^{-g_{i}\left(x_{n},c\right)}} \end{array}$$

In the formula, $g_{i}\left(x_{n},c\right)$ is the computed result of a linear transformation where c∈{0,1}. The sigmoid function maps the input values to the range [0,1], representing the probability of the sample belonging to class 0 or 1. The expression for the MSE loss function is as follows.

(4) $$\begin{array}{c} L_{MSE}=\frac{1}{n}\sum_{i=1}^{n}{\left(p_{i}-y_{i}\right)}^{2} \end{array}$$

In the above formula, $y_{i}$ represents the label of sample *i*, while $p_{i}$ represents the predicted probability of sample *i* belonging to that label.

### 2.3. Individual Difference Module

**Figure 6:**
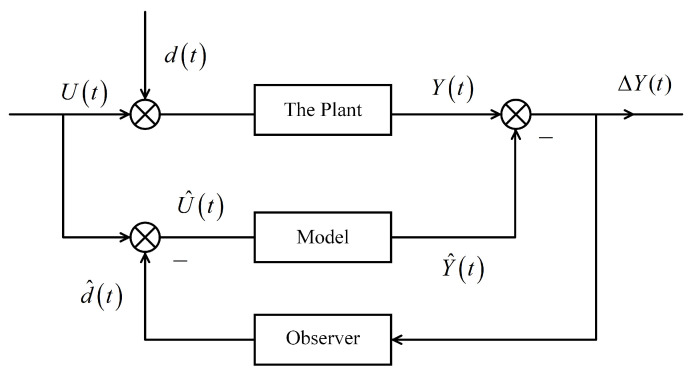
IDM with distribution.

The IDM is designed to observe and eliminate individual differences. In the task of emotion recognition, individuals may exhibit variations in their responses to emotions. While the emotion classification module can classify the overall data, it cannot provide sufficiently accurate predictions for individual differences. Therefore, the purpose of the IDM is to calibrate the output of the emotion classification module in order to predict individual emotions more accurately. The goal of this model is to reduce the interference caused by individual differences, thereby approaching the accuracy of an ideal model. Refer to Figure 6 for further details.

We denote the input of the model as $U_{i}\left(t\right)$ and the interference related to individual differences as $d_{i}\left(t\right)$; then, the input with interference is ${\hat{U}}_{i}\left(t\right)=U_{i}\left(t\right)+d_{i}\left(t\right)$, the ideal output is $Y_{i}\left(t\right)$, and the output of the trained model is ${\hat{Y}}_{i}\left(t\right)$, where *i* denotes the EEG signal of the *i*th sample. The error of the output is denoted as $\Delta Y_{i}\left(t\right)=Y_{i}\left(t\right)-{\hat{Y}}_{i}\left(t\right)$. To introduce observation, we incorporate an equivalent interference ${\hat{d}}_{i}\left(t\right)$ through observation, aiming to make $d_{i}\left(t\right)-{\hat{d}}_{i}\left(t\right)\to 0$ and $\Delta Y_{i}\to 0$. Because the model requires a large number of samples, when all samples are used as the inputs $U\left(t\right)=\sum_{i=1}^{n}U_{i}\left(t\right)$ the interference $d\left(t\right)=\sum_{i=1}^{n}d_{i}\left(t\right)$ follows a Gaussian distribution, specifically white noise, which means that d(t)=0 and $U\left(t\right)=\hat{U}\left(t\right)$. The objective of training the model is to minimize the difference between the output of the trained model and the ideal model, i.e., ΔY→0.

In this module, “the plant” refers to the neural mechanism from the participants, representing a physical entity, while the “model” represents an emotion classification model without individual differences, which is a essentially universal emotion recognition model. It takes the EEG signals of participants as input and apply deep learning techniques to minimize any discrepancies output between the training model and the ideal model in the absence of individual differences. Specifically, the output generated by the training model is almost identical to the output of the ideal model.

In fact, “the plant” cannot ignore the individual differences, which are denoted as d(t). Individual differences can bring about errors in the model output, potentially affecting the recognized classification result $\hat{Y}\left(t\right)$. In order to obtain an accurate $\hat{Y}\left(t\right)$, an observer is designed to observe and compensate for this difference, bringing the output closer to the actual situation of the individual.

The observer compares the actual output with the expected output, which generates an error signal indicating the difference between the expected output and the actual output. The observer uses error signals to adjust the behavior of the system, then generates an adjusted output signal based on the correction measures calculated in the previous step. The adjusted output is sent back to the feedback loop as the output of the system. The above process is iterative; as the system continues to operate, the observer continuously monitors errors and adjusts the output to reduce errors over time.

We selected the isotonic regression algorithm [32] to obtain the observer effect. By taking the output of the emotion classification module as input to the isotonic regression module, the monotonicity of the original classification results can be preserved. Furthermore, by appropriately adjusting the output based on individual differences, the model can better reflect the individual’s emotional state, thereby enhancing the accuracy of the model at the individual level. The advantage of isotonic regression lies in its independence from any specific functional form. Instead, it determines the shape of the model based on the ordering relationship within the data itself. Therefore, it is suitable for various types of data. The detailed mathematical definition of isotonic regression provided below.

Consider the original training set of model $\left(x_{m},y_{m}\right)$, where $x_{m}\in X$ and $y_{m}\in Y$ are the prediction probabilities and class labels, respectively, and m=1,2,…,M denotes the *m*th sample. Define the model to be calibrated as $f_{m}=f\left(x_{m}\right)$ with the training set $\left(f_{m},y_{m}\right)$; then, the isotonic regression problem is to find an isotonic regression function ${\hat{y}}_{i}$ such that

(5) $$\begin{array}{c} y_{m}^{*}={\mathrm{argmin}}_{\hat{y}}\sum_{m=1}^{M}\omega_{m}{\left[y_{m}-{\hat{y}}_{m}\left(f_{m}\right)\right]}^{2} \end{array}$$

where $\omega_{m}>0$ is a weight coefficient and ${\hat{y}}_{m}$ is monotonically increasing and non-parametric. It should be noted that the above theory is established following the assumption that

(6) $$\begin{array}{c} y_{m}=\hat{y}\left(f_{m}\right)+\varepsilon_{m} \end{array}$$

where $\varepsilon_{m}$ is the error.

Finally, the obtained model is used to adjust the emotion classification results of the general model, thereby obtaining more accurate individual emotion recognition outcomes.

## 3. Model Implementation

In the IERM, the input data first pass through four convolutional layers, which are sequentially combined to form the CNN module. Each convolutional layer consists of a batch normalization layer, an ECA layer, a dropout layer, and an activation layer. The activation layer selects the leaky rectified linear unit (ReLU) function [33]. By stacking and combining these layers, the model can extract useful features from the input images, while the feature representation capability is enhanced through the ECA mechanism and dropout layer. The leaky ReLU layer helps the model to learn more complex feature representations. At the same time, a max pooling operation and data flattening are applied to reduce the impact of irrelevant information and transform multidimensional inputs into one-dimensional form, facilitating smooth transition of data from the convolutional layers to the fully connected layers. The processed data are then fed into the BiLSTM layer, which can better capture contextual features in the sequence by simultaneously processing forward and backward information, thereby enhancing the model’s representation capability and performance. The hidden states and memory states of the model are updated in the BiLSTM layer using a hidden layer size of 128. During the training process, the optimizer uses the adam algorithm [34]. The adam algorithm combines the characteristics of momentum and adaptive learning rates, allowing for adaptive adjustment of the learning rate based on the gradients of different parameters. In addition, we define the IERM using the MSE loss function as the loss function. The MSE loss penalizes model errors, making the model pay more attention to samples with larger errors, which improves the prediction accuracy of the model for such samples.

The IDM utilizes isotonic regression to observe and calibrate the output of the model. To solve the isotonic regression problem, the pair-adjacent violators (PAV) algorithm [35] is used, as it provides a stepping constant solution. The specific method is as follows. For $Y=(y_{1},y_{2},\cdots ,y_{M})$, let $Y^{*}=\left(y_{1}^{*},y_{2}^{*},\cdots ,y_{m-1}^{*},y_{m},\cdots ,y_{M}\right)$ if

(7) $$\begin{array}{c} y_{m}-y_{m+1}\leq 0 \end{array}$$

meaning that $y_{m}^{*}=y_{m}$; otherwise, if

(8) $$\begin{array}{cc} y_{m}-y_{m+1} & >0\\ y_{m-1}^{*}-y_{m+1} & >0\\ \; & \vdots \\ y_{l}^{*}-y_{m+1} & >0\\ y_{l-1}^{*}-y_{m+1} & \leq 0 \end{array}$$

then $y_{l}^{*}=\cdots =y_{m}^{*}=y_{m+1}^{*}=\frac{\sum_{u=l}^{m+1}\omega_{u}y_{u}}{\sum_{u=l}^{m+1}\omega_{u}}$, where l∈{1,2,⋯,m}.

## 4. Experiment and Analysis

This section introduces the widely used DEAP database [23]. The implementation of the proposed model is demonstrated through experiments using DEAP, and the experimental results obtained using this approach are presented along with a corresponding analysis.

### 4.1. Dataset and Preprocessing

The DEAP dataset comprises EEG signals obtained from a diverse group of 32 participants, including an equal distribution of 16 males and 16 females, all of whom were carefully selected to ensure a healthy physiological and psychological state. Participant recruitment involved outreach through academic institutions and community networks. Each participant voluntarily participated in the study after providing informed consent. For EEG signal acquisition, we utilized a 32-channel electrode cap in compliance with the international standard for electrode placement. To maintain consistency and capture emotional responses, participants were exposed to 40 distinct one-minute music videos as stimuli. These videos were intentionally chosen to evoke a range of emotions, and participants were instructed to engage with each video naturally. Integrally to the experiment, participants were requested to complete a self-assessment manikin (SAM) [36,37] questionnaire after each trial. The SAM questionnaire is characterized by its rapid and simple measurement attributes, and assesses three dimensions of emotion (Pleasure, Arousal, Dominance) in a non-verbal manner. The questionnaire asked participants to rate the levels of arousal and valence they experienced while viewing the music video stimuli using a scale ranging from 1 (low) to 9 (high). Further details pertaining to the dataset are succinctly summarized in Table 1.

During the data preprocessing stage, blind source separation techniques were employed to remove the electro-oculogram (EOG) from the EEG signals. Additionally, the baseline data from the first 3 s of each trial were discarded. Subsequently, the data underwent downsampling to reduce the sampling frequency to 128 Hz. In order to focus on the frequency bands associated with emotions, bandpass filtering was applied to the signals in four frequency bands (θ, α, β, γ). In this experiment, we selected valence and arousal levels as the criteria for emotional assessment. Based on the levels of arousal and valence, the trials were categorized into two classes, with 0 representing negative emotions and 1 representing positive emotions. The threshold was set at 5, with ratings ranging from 1 to 5 classified as negative emotions and ratings from 6 to 9 classified as positive emotions.

### 4.2. Experimental Design

The dataset of 32 individuals was divided into training set, test set, and cross-subject dataset in a ratio of 0.6, 0.2, and 0.2, respectively. Therefore, the training set should consist of 19 samples, while the test set and cross-subject dataset should each contain six samples. The training process involves two stages. In the first stage, the model for group-based emotion recognition is trained. This module relies on the data from all the participants to learn the patterns of emotion recognition at the group level. In the second stage, the six samples from the cross-subject dataset are fed into the model that incorporates the IDM. The purpose of this module is to capture individual variations by calibrating the model’s output, thereby improving the accuracy of individual emotion recognition. The model was trained for 100 epochs with a learning rate of 0.001. To address overfitting, a dropout value of 0.5 was used. For the dataset, ten-fold cross-validation was employed, and the model’s performance was evaluated by averaging the accuracy across all participants.

### 4.3. Comparative Experiment

To evaluate the effectiveness of the group emotion recognition model, we conducted extensive experiments on the dataset. Three neural network models were designed, including a CNN, 3D Convolutional Neural Network (Conv3D) [38], and IERM without IDM. The emotion recognition module of IERM consists of CNN and LSTM, aiming to integrate both temporal and spatial features in the time-frequency domain.

We first attempted the CNN model, which effectively captures spatial correlation features in EEG signals through a combination of convolutional and pooling layers [39,40]. The convolutional layers automatically learn patterns at different frequencies, while the pooling layers reduce dimensionality and extract the most important features. CNN directly extracts features from raw EEG signals and feeds the extracted features into a classifier for emotion classification. Conv3D extends CNN to three-dimensional space [41]. It can handle EEG signal data that include a temporal dimension. By performing convolutions along the temporal dimension, Conv3D captures the temporal sequence features of EEG signals, providing a more comprehensive representation of the dynamic changes in EEG signals. The IERM model is combined with CNN and BiLSTM to handle EEG-based emotion recognition tasks. The CNN is used to extract spatial features, incorporating the ECA mechanism to enhance the network’s focus on key channels for emotion classification. BiLSTM, on the other hand, is employed to process the temporal sequence information. The model can effectively integrate spatial and temporal features of EEG signals, thereby strengthening the representation capacity of important channels and achieving more accurate emotion recognition.

In the CNN model, the input data undergo convolution operations and nonlinear transformations through the ReLU activation function, then pass through four convolutional layers and are downsampled by the max-pooling layer. Next, the data are flattened into a one-dimensional vector and input into fully connected layers. The output of the fully connected layers is nonlinearly transformed using the ReLU activation function and then passed to the output layer. Finally, the model returns the output results. In the Conv3D model, the preprocessed data are transformed from two-dimensional to three-dimensional data. This model includes four 3D convolutional layers and two fully connected layers. The input data have a dimensionality of five. The model extracts features using 3D convolutional and pooling layers and performs classification through the fully connected layers, ultimately outputting the classification results. The IERM (excluding the IDM) has the specific network structure mentioned earlier. In the emotion classification module of IERM, we initially consider a version using the softmax function as the activation function for the output nodes and employing cross-entropy as the loss function [42].

Furthermore, we treat this binary classification problem as a special case of regression. We consider a threshold classifier (sigmoid function) to convert probabilities into the final binary labels, and employ the MSE as the loss function.

Table 2 presents the recognition accuracy of these models on the DEAP dataset. It is evident that the emotion classification module utilizing the MSE loss function achieves higher accuracy on the test set. Therefore, we selected this module as the emotion classification component for our overall model. Additionally, the table indicates a noticeable decrease in accuracy for cross-subject emotion classification, as shown by our partitioning.

**Figure 7:**
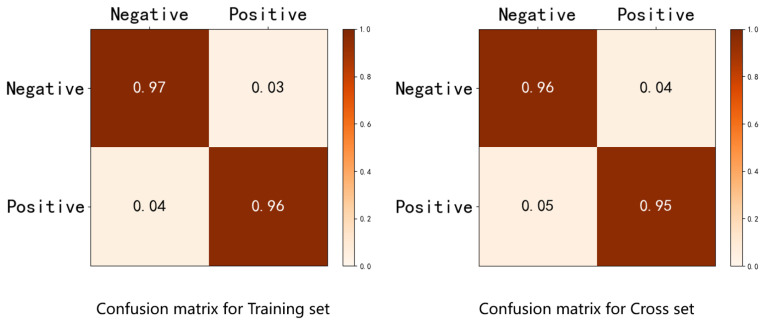
Confusion matrix comparing the training set and cross set.

The confusion matrix visually represents the algorithms’ performance, which facilitates the identification of classification errors. It represents the most fundamental and intuitive method of evaluating the accuracy of classification models. The confusion matrix is a square matrix of size n×n, where *n* represents the number of classification categories; each row represents the predicted values and each column represents the true values. Larger values on the diagonal indicate superior performance of the corresponding model. The recognition accuracy of IERM is depicted using confusion matrices for visual representation of the comparative results in Figure 7, with MSE as the performance metric.

**Figure 8:**
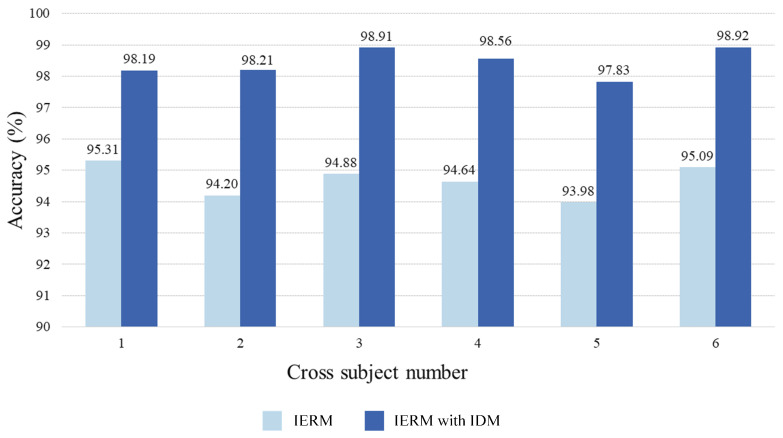
Comparison of the average cross-subject accuracy between IERM and IERM with IDM.

We fed the six cross-subject datasets into the IERM with the IDM. Table 3 provides the recognition accuracy of these six cross-subject classifications with and without the IDM. Figure 8 demonstrates that the presence of the IDM significantly improves the cross-subject classification results, with an improvement ranging from 2.8% to 4.0% compared to the results without the IDM.

The highest classification accuracy achieved among the six cross-subject classifications is 98.92%. In reference [22], the researchers introduced an approach that combines an individual similarity-guided transfer modeling framework with transfer learning. This approach takes into account individual differences that disrupt the assumption of identical distributions. On the DEAP dataset, this method achieved accuracies of 66.1% for valence and 66.7% for the arousal dimension. In [21], the authors reduced the number of EEG electrode channels and proposed an experiment-level batch normalization method to mitigate the negative impact of individual differences. The recognition accuracy reached up to 89.63%. Regarding personalized EEG signal emotion recognition, in [43] the authors categorized individuals based on their personality traits and then utilized a deep learning to model the spatiotemporal features of EEG signals. This personalized recognition approach achieved an accuracy of 72.4% for valence and 75.9% for the arousal dimension. In [44], a method was proposed combining Multi-Scale Residual Network (MSRN) with a Meta-Transfer Learning (MTL) strategy for EEG-based emotion recognition. This approach reduced the problem of individual differences between subjects, yielding an accuracy of 71.29% for valence and 71.92% for the arousal dimension on the DEAP dataset. The emotion recognition method proposed in the present study surpasses the recognition accuracy of the aforementioned research methods. The significant improvement in accuracy effectively highlights the superiority of the approach presented in this paper.

## 5. Conclusions

The proposed IERM combines time–frequency and spatial domain features, incorporating a channel attention mechanism. By learning the inter-channel correlations and adaptively adjusting their weights, the network’s performance is enhanced. Furthermore, an individual differences module is introduced to fine-tune the accuracy of emotion recognition results for each participant, enabling the model to better accommodate the distinct EEG signal characteristics and minimize individual variations in emotion recognition. The proposed method achieves high accuracy in emotion classification and outperforms existing models, with the highest accuracy rates of 96.43% and 98.92% for population-based and cross-participant scenarios, respectively, based on the DEAP dataset. The introduction of this model enhances the details and content of emotion recognition research and yields improved performance, especially in scenarios characterized by significant individual differences.

In our subsequent work, we intend to explore ways to further enhance estimation methods for individual differences while considering the optimization of existing models. This exploration will involve expanding the dataset along with other approaches to further investigate the method proposed in this paper while considering the practical application of the model.

## Figures and Tables

**Table 1:** Description of the dataset.

Type	Description
subject	32 (16 males and 16 females)
channels	32
sampling rate	32 (128 Hz)
moviest	40 different movie clips
data shape	(40, 32, 8064)

**Table 2:** Accuracy comparison between the different models.

Model	Training Set	Cross Subject
CNN	67.57%	65.02%
Conv3D	82.45%	78.69%
IERM (Cross Entropy)	93.86%	91.42%
IERM (MSE)	96.43%	95.31%

**Table 3:** The comparison of accuracy with and without IDM.

Cross Subject Number	IERM	IERM with IDM
1	95.31%	98.19%
2	94.20%	98.21%
3	94.88%	98.91%
4	94.64%	98.56%
5	93.98%	97.83%
6	95.09%	98.92%

## Data Availability

Research studies the publicly available DEAP dataset, please refer to: http://www.eecs.qmul.ac.uk/mmv/datasets/deap/download.html.
